# Engineering Au single-atom sites embedded in TiO_2_ nanostructures for boosting photocatalytic methane oxidation†

**DOI:** 10.1039/d4na00947a

**Published:** 2025-01-13

**Authors:** Qui Thanh Hoai Ta, Ly Tan Nhiem

**Affiliations:** a Institute of Chemical Technology, Vietnam Academy of Science and Technology 1A TL29 Street, Thanh Loc Ward, District 12 Ho Chi Minh City 700000 Vietnam tathanhhoaiqui2292@gmail.com; b Faculty of Chemical and Food Technology, Ho Chi Minh City University of Technology and Education 01 Vo Van Ngan Street, Linh Chieu Ward, Thu Duc City Ho Chi Minh City 700000 Vietnam nhiemlt@hcmute.edu.vn

## Abstract

Photocatalytic methane oxidation under mild conditions using single-atom catalysts remains an advanced technology. In this work, gold single atoms (Au SAs) were introduced onto TiO_2_ nanostructures using a simple method. The resulting performance demonstrated effective conversion of methane into H_2_ and C_2_ products at room temperature. The as-synthesized Au SA/TiO_2_ exhibited a high hydrogen production rate of 2190 μmol g^−1^, with selectivity reaching up to 58% under optimized conditions. The methane oxidation mechanism was investigated, revealing a methyl radical pathway for generating value-added chemicals. This research provides a strategy for photocatalytic methane conversion over single-atom-supported photocatalysts.

## Abstract 2

Photocatalytic methane oxidation under mild conditions using single-atom catalysts remains an advanced technology.

## Graphical abstract

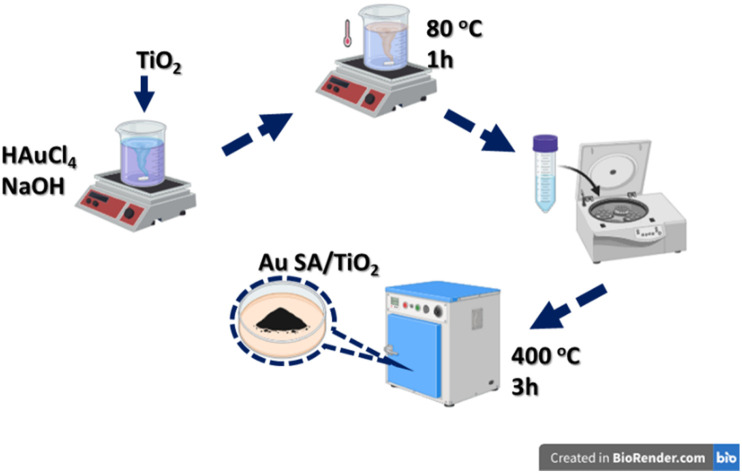

## 1. Introduction

Methane (CH_4_) is a crucial fossil fuel and an important component of natural resources, serving as a significant chemical raw material. It constitutes approximately 80% of natural gas, making it a massive contributor to the greenhouse effect and global warming, particularly during the combustion process compared to carbon dioxide (CO_2_).^1,2^ Therefore, the sustainable harvesting of CH_4_ and its conversion into valuable products is essential for the sustainable utilization of natural resources and environmental protection. However, conventional methods for CH_4_ conversion typically require high operating conditions (>100 °C and 100 MPa), which can result in excessive oxidation and low product selectivity. This is due to the bond dissociation energy of the initial C–H bonds being higher than that of the subsequent C–H bonds, which prevents the activation of the first C–H bond when relying solely on thermal energy to overcome the activation barrier.^3–5^ Thus, it remains a challenge to enhance the selectivity of CH_4_ conversion to high-value-added products under mild conditions.^6,7^ Photocatalysis has been recognized as a green technique and a unique method for harnessing light to generate electron–hole pairs in semiconducting materials.^8–11^ The application of single-atom catalysts in methane oxidation shows significant advancement over traditional catalysts, as they exhibit enhanced catalytic performance by uniformly decorating noble metal atoms. This arrangement facilitates active exposure and increases the surface area for the oxidation process. Additionally, single-atom catalysts reduce the amount of catalyst required, addressing concerns about high costs and the scarcity of precious metals, making them economically viable even in small quantities.^12–14^ It is crucial to understand the overall reaction rate of photocatalysts with varying active atom loadings due to their low coverage, which helps prevent the formation of nanoparticles. Single-atom catalysts also integrate seamlessly with advanced nanomaterials, such as reduced graphene oxide and carbon nanotubes, further improving catalytic activity and stability. These catalysts have demonstrated the ability to reduce dependence on noble metals while enhancing selectivity, owing to the abundance of active sites. Several studies have focused on engineering noble metal atoms (*e.g.*, Pt and Ag) on titania catalysts, exploring the role of titanium cations and oxygen vacancies in the localization of single atoms on oxo ligands, thereby facilitating superior photocatalytic performance.^15–20^

Titanium dioxide (TiO_2_) has garnered significant attention within the scientific community and has become a prominent commercial product due to its unique properties and diverse applications. Its excellent chemical stability, strong catalytic ability, and wide band gap energy (*E*_g_) under light illumination contribute to its utility.^21–23^ The application of TiO_2_ as a catalyst in photocatalytic methane oxidation and water splitting presents a promising solution in response to the urgent demand for net-zero emissions and sustainable energy applications. However, one of the main disadvantages of TiO_2_ is its wide band gap and the rapid recombination rate of electron–hole pairs, which poses challenges for researchers. Numerous strategies have been explored to enhance the photocatalytic performance of TiO_2_, including metal doping, non-metal loading, and surface modification with other semiconductors or quantum dots.^3,24,25^ These combinatorial techniques aim to reduce the electron–hole recombination rate, extend light harvesting capabilities, and improve surface reaction kinetics.

Building on this foundation, the highly selective methane oxidation using an Fe/TiO_2_ catalyst was investigated in 2018, which demonstrated a 15% conversion rate after 3 hours of reaction, with methanol selectivity exceeding 90%. The presence of FeOOH and Fe_2_O_3_ plays a crucial role in enhancing charge transfer and separation in TiO_2_, thereby reducing the overall overpotential of the reaction. Photocatalytic methane oxidation over the TiO_2_ nanostructure is commonly considered owing to its low cost, stable chemical properties, and non-toxic nature.^26^

In the same context, photocatalytic CH_4_ oxidation to C_1_ oxygenates over Pd/TiO_2_ composites was investigated using O_2_ and H_2_O as oxidants. Palladium (Pd) serves as a hole acceptor, while oxygen vacancies function as electron acceptors, enhancing charge separation efficiency. Under optimized conditions, C_1_ products were obtained with approximately 99% selectivity and a high yield of 54.7 mmol g^−1^ h^−1^.^27^ Shuang *et al.* utilized Au–ZnO/TiO_2_ composites for the direct oxidation of CH_4_ to valuable C_2_H_6_ in a flow reactor under mild conditions. A C_2_H_6_ production rate of over 5 mmol g^−1^ h^−1^ was achieved after 50 minutes of reaction, with a high selectivity of 90% attributed to the weak overoxidation ability of ZnO. In this system, Au facilitates the desorption of ˙CH_3_ radicals in the gas phase, thereby preventing overoxidation to CO_2_.^28^

In this work, Au single-atom (Au SA) supported TiO_2_ nanostructures were investigated for driving CH_4_ oxidation under mild conditions. Due to energetic active sites of Au SAs, the optimized production yield of H_2_ reached 2190 μmol g^−1^, with a selectivity of 58%. The investigation into the plausible mechanism indicated that Au inhibits the charge recombination rate and activates the reactant gases. These results suggest that this approach can effectively enhance H_2_ production through improved photocatalytic performance at room temperature.

## 2. Experimental

### 2.1. Materials

Titania powder (TiO_2_, P25, 99%), gold(iii) chloride tetrahydrate (HAuCl_4_·4H_2_O), sodium borohydride powder (NaBH_4_, 98%), and sodium hydroxide (NaOH) were purchased from Sigma-Aldrich. Deionized (DI) water and ethanol (C_2_H_5_OH) were used as a solvent for material cleaning steps. All chemicals were used without further purification.

### 2.2. Preparation of catalysts

**Fig. 1:**
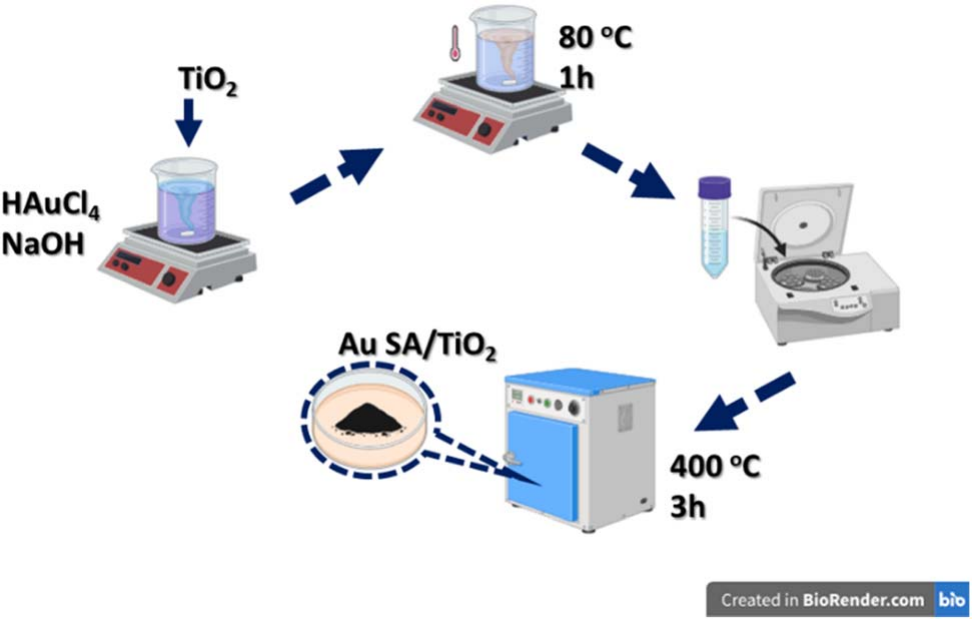
The schematic illustration of Au single-atom (Au SA) – supported catalysts.

The Au SA-supported photocatalysts were synthesized using a wet precipitation process, as presented in Fig. 1. Specifically, 0.3 g of TiO_2_ was added to an aqueous solution of HAuCl_4_ (1.5 mg mL^−1^), with the pH pre-adjusted to approximately 9 using 0.1 M NaOH solution and NaBH_4_ solution, followed by stirring at 80 °C for 1 hour. After the reaction, the precipitates were collected, thoroughly washed with deionized water, and dried at 60 °C overnight. The loading content of Au SAs was estimated at 2 wt% as the standard dose. The final product was calcined at 400 °C for 3 hours, ground, and denoted as Au SA/TiO_2_, which was then stored for subsequent photocatalytic experiments. For comparison, Au NPs/TiO_2_ and Pt NPs/TiO_2_ were also prepared *via* the traditional impregnation technique without pH control, as in previous publications.^29,30^

### 2.3. Characterization

The crystal structure of the prepared photocatalysts and pure TiO_2_ was examined by using an X-ray diffractometer (XRD), equipped with a Cu-Kα radiation source working at 40 kV and 30 mA. The presence of single atoms was observed by using a scanning transmission electron microscope (S/TEM) equipped with an HAADF detector (electron energy of 300 keV). The optical absorption and radiative recombination of photocatalysts were studied by ultraviolet-visible diffuse reflectance spectroscopy (UV-vis DRS) and photoluminescence (PL) using a spectrometer (PerkinElmer Lambda 650S) and HR Labram, Horiba, respectively. The optical properties of selected samples were confirmed by using Fourier transform infrared spectroscopy (FTIR/JASCO-4600), Raman spectroscopy (Olympus-BX5), a semiconductor device analyzer (Keysight B1500A), and a CH instruments electrochemical workstation.

The analysis of paramagnetic species was performed by continuous-wave electron paramagnetic resonance (EPR). These experiments were performed on a Bruker ELEXSYS E500 spectrometer operating in the X-band (9.5 GHz). The EPR spectra were recorded at 94 K to avoid electron–hole recombination. The detection of radical species was conducted by spin-trapping tests using 5-*tert*-butoxycarbonyl-5-methyl-1-pyrroline-*N*-oxide (BMPO) as the scavenger. The atomic-scale and electronic structure of the sample were investigated by scanning tunnelling microscopy (STM) measurement. For this, the Au SA/TiO_2_ solution was drop-cast on a gold plate, that was quickly loaded in the load lock chamber of a UHV system (base pressure < 1 × 10^−10^ Torr) to be heated for twelve hours at 85 °C. The STM experiments were performed at room temperature with a tungsten tip and under tunneling conditions: *V*_sample_ = 1.8 V, *I*_t_ = 200 pA.

### 2.4. Photocatalytic methane oxidation

The photocatalytic oxidation of CH_4_ by CO_2_ molecules was carried out in a batch stainless-steel reactor (100 mL), with a circular quartz window attached on top. In brief, 50 mg of the sample was placed in the reactor, which was closed and evacuated for 10 min prior to the provision of 10 bars of the CH_4_ : CO_2_ gas mixture (3 : 1 pressure ratio) and kept under dark conditions for 30 min. Afterwards, a 300 W xenon lamp (200–1000 nm) was switched on as the light source. The wavelength of light irradiation could be varied by specific filters. The chamber was kept at room temperature by using a chiller. The gaseous products were analyzed and quantified using a gas chromatography system (HP-6890, Agilent).

## 3. Results and discussion

### 3.1. Structural and morphological properties

**Fig. 2:**
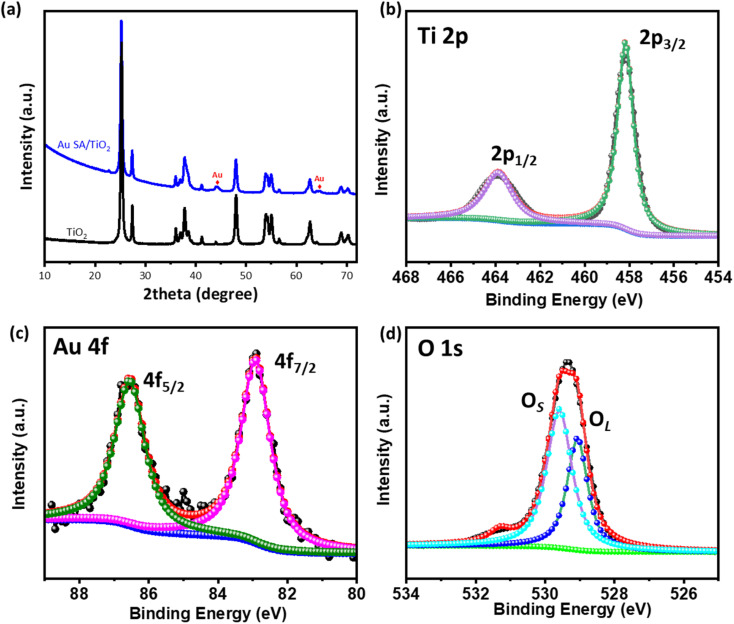
(a) XRD patterns of pristine TiO_2_ and the Au SA/TiO_2_ composite and (b)–(d) XPS spectra of Ti 2p, Au 4f, and O 1s of the selected Au SA/TiO_2_ composite.

The crystal structures of the samples were characterized by X-ray diffraction (XRD). As shown in Fig. 2a, the TiO_2_ nanostructures exhibit mixed crystal phases of rutile and anatase, corresponding to the standard JCPDS data (no. 21-1272 for rutile and no. 21-1276 for anatase).^31–33^ Upon calcination at 400 °C, the well-defined diffraction peaks at 25.8°, 28.1°, 37.1°, 48.3°, and 55.3° were assigned to the (101), (110), (004), (220), and (105) planes of TiO_2_, respectively. Additionally, the broadened peaks at 44.1° and 64.1° were observed, indicating the presence of Au SAs on the TiO_2_ nanostructures. The relatively small size of the Au diffraction peaks suggests high dispersion of the Au SA. Fig. 2b–d illustrate the Ti 2p, Au 4f, and O 1s region spectra of the selected Au SA/TiO_2_ composite. The main photoelectron peaks at binding energies of 463.8 eV and 458.3 eV are assigned to Ti^4+^ 2p_1/2_ and 2p_3/2_, respectively (Fig. 2b).^34^ The Au 4f spectra were deconvoluted into two major peaks at 86.5 eV and 82.9 eV, corresponding to the characteristic peaks of Au 4f_5/2_ and Au 4f_7/2_, respectively (Fig. 2c). These findings suggest that Au is reduced from Au^3+^ to Au^1+^ upon the formation of single atoms.^35,36^ As observed in Fig. 2d, the O 1s peak at 529.4 eV can be deconvoluted into two peaks, with primary peaks at 529.1 eV and 529.6 eV corresponding to lattice oxygen (O_L_) and surface-adsorbed oxygenated species (OH_s_/O_s_), respectively.^34^ This confirms the creation of oxygen vacancies. These characterization studies confirm the phase and surface chemical states of the Au SA/TiO_2_ catalyst.

**Fig. 3:**
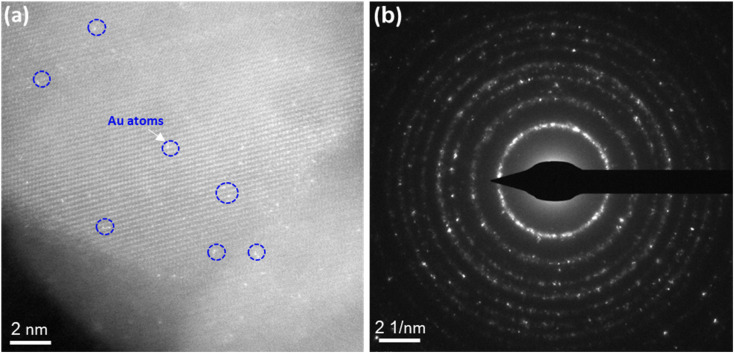
The HR-TEM image and SAED images of Au SA/TiO_2_ powder, respectively (a) and (b). The bright spots in (a) indicate Au single atoms (SA) over the TiO_2_ matrix.

Due to the inherent resolution limitations of the SEM system, these images cannot definitively confirm the formation of single atoms or nanoparticle assemblies. Therefore, high-resolution TEM (HR-TEM) analysis provides clear evidence of the distribution of Au SAs on the surface of TiO_2_ nanostructures. As illustrated in Fig. 3, the Au SAs are atomically dispersed across the TiO_2_ surface, visible through their brighter contrast (highlighted by the blue circle). This confirms the enhanced distribution of Au SAs as a result of the calcination process, with no noticeable agglomeration compared to the Au NPs/TiO_2_ synthesized *via* traditional impregnation methods. In other words, TiO_2_, as a reducible oxide, is well-suited for the atomic dispersion of metal catalysts due to its inherent surface defects, which help stabilize metal atoms by forming metal–oxygen-support bonds.^37,38^

**Fig. 4:**
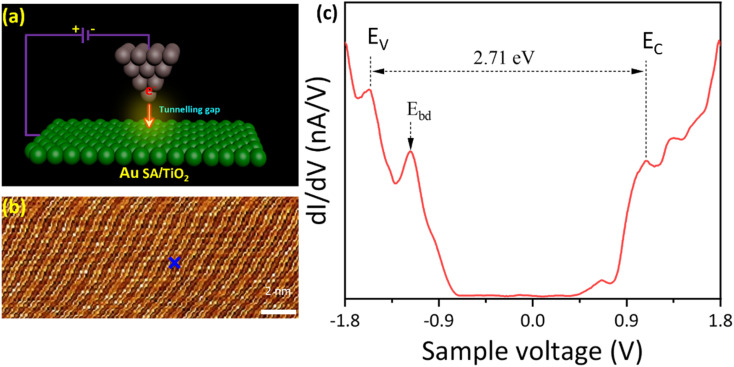
(a) The schematic illustration of scanning tunneling microscopy (STM) analysis, (b) and (c) topography STM image and tunneling spectroscopy of the Au SA/TiO_2_ sample recorded from the green cross in (b). Tunneling conditions: *V*_sample_ = 1.8 V, *I*_t_ = 200 pA. The valence and conduction band edges of Au SA/TiO_2_ are indicated by vertical segments, labeled as *E*_V_ and *E*_C_. The *E*_bd_ stands for bright defects in the TiO_2_ structure.

To further investigate the Au SA/TiO_2_ heterostructure at the atomic scale, the STM analysis has been carried out (schematized in Fig. 4a). The topography STM image indicated the atomic arrangement of TiO_2_ powder, although the signals of Au SAs were unable to be detected (Fig. 4b). By selecting a specific area on the catalyst surface, STM measurement was conducted and revealed the electronic structure of Au/TiO_2_ (Fig. 4c). By measuring the distance between peaks of the valence band (*E*_V_) and conduction band (*E*_C_), the bandgap energy of Au SA/TiO_2_ was estimated to be 2.71 eV, which is smaller than 3.2 eV of pristine TiO_2_ P25, leading to favorable benefits for photocatalytic reactions.^39^ Besides, the bright defects (*E*_bd_) near the valence band were also observed, indicating shallow trapping states of TiO_2_ crystals.

### 3.2. Optical properties

**Fig. 5:**
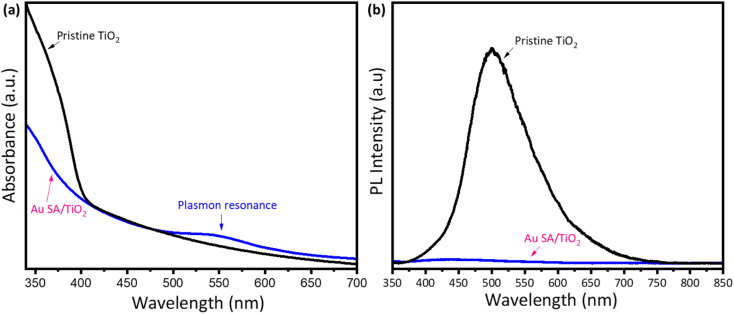
The UV-vis absorption spectra of pristine TiO_2_ and Au SA/TiO_2_, respectively (a) and (b).

To understand the effect of Au loading on the light absorption properties of TiO_2_, UV-vis spectra were analyzed. As shown in Fig. 5a, apart from strong absorption of pristine TiO_2_ below 400 nm, the Au SA/TiO_2_ powder exhibited another absorption peak at 550 nm, indicating the plasmonic effects, which were absent in the case of the pristine TiO_2_ spectrum. The plasmon resonance of Au SAs could significantly contribute to photocatalytic performance later. Moreover, the steady-state photoluminescence (PL) spectra were obtained to confirm the separation properties of photogenerated carriers (Fig. 5b). The pure TiO_2_ samples exhibited stronger photoluminescence emission signals compared to Au SA/TiO_2_, indicating a higher recombination rate of electrons and holes. Notably, Au SA/TiO_2_ displayed the lowest fluorescence intensity, suggesting efficient separation of photocarriers. This finding indicates that the transfer of excited electrons occurred rapidly between Au and TiO_2_, effectively reducing the recombination rate in the photocatalysts.^40,41^ The photocatalysts were analyzed using the photocurrent response and electrochemical impedance spectroscopy. Fig. S1a† displays the current–voltage (*I*–*V*) curve for the Au SA/TiO_2_ photocatalyst under both dark and light conditions, with the applied voltage ranging from −1 V to +1 V. Under light illumination, the slope of the *I*–*V* curve begins to increase, indicating that the generated charge carriers significantly influence the electrical behavior of the optimized sample. Furthermore, the Nyquist plot in Fig. S1b† shows that the electron transport capability of the electrode surface has been slightly enhanced in the Au SA/TiO_2_ sample compared to pure TiO_2_.

**Fig. 6:**
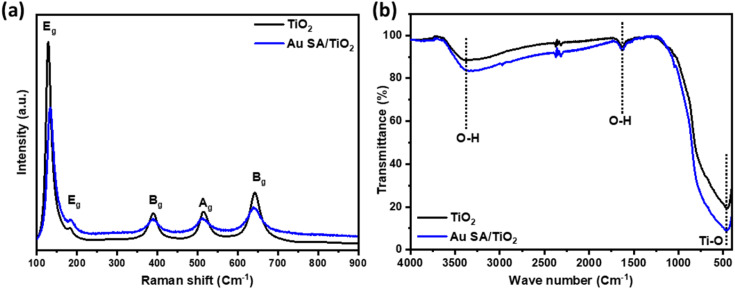
(a) The Raman and (b) FTIR spectra of pristine TiO_2_ and Au SA/TiO_2_, respectively.

The interaction between TiO_2_ and Au SAs was analyzed using Raman spectroscopy and FTIR. As shown in Fig. 6a and S2a,† the Raman spectrum of the selected samples displays five characteristic modes located at 128, 182, 390, 514, and 643 cm^−1^, which correspond to the E_g_, E_g_, B_g_, A_g_, and E_g_ modes, respectively. The main peak at 128 cm^−1^, characteristic of the Ti–O stretching mode in pristine TiO_2_, positively shifts to 133 cm^−1^, confirming that Au has interacted with the Ti–O bond. This shift indicates the strengthening of the Ti–O bond, as well as phonon confinement effects associated with the creation of oxygen vacancies.^42,43^ The FTIR spectra of all samples show bands corresponding to TiO_2_, with a broad band from 400 to 900 cm^−1^ assigned to Ti–O–Ti vibrations, as well as bands at around 1621 cm^−1^ and 3416 cm^−1^, which are attributed to O–H bending and O–H stretching vibrations, respectively (Fig. 6b and S2b†).^44,45^ The FTIR spectrum of TiO_2_ after modification with Au does not exhibit significant changes compared to pristine TiO_2_, likely due to the formation of Au single atoms on the overall TiO_2_ structure.

### 3.3. Photocatalytic performance

**Fig. 7:**
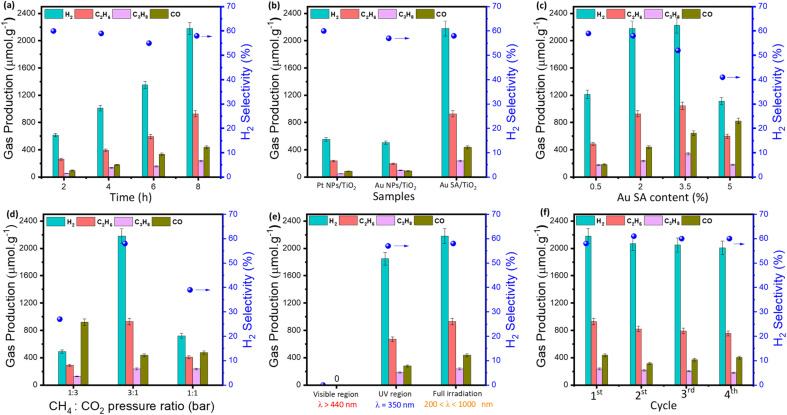
Photocatalytic methane oxidation performances and the H_2_ selectivity as a function of reaction time over the standard Au SA/TiO_2_ sample (2 wt% Au) (a), comparable photocatalytic experiments of different catalysts (b), comparison of photocatalytic activity with different Au loading contents, reactant ratios, and irradiation regions, respectively (c)–(e), and stability tests over four cycles (f). Reaction conditions: 50 mg of the standard Au SA/TiO_2_ catalyst with 2% Au loading, CH_4_ : CO_2_ = 3 : 1 (stand pressure ratio), xenon lamp 300 W. In figures (a), (d) and (f), standard Au SA/TiO_2_ was used.

According to unique properties, the Au SA/TiO_2_ sample was employed for photocatalytic CH_4_ oxidation by CO_2_ molecules, as depicted in Fig. 7. Note that there were no products generated in the absence of catalysts and light irradiation (Fig. S3†). By contrast, under light conditions, the Au SA/TiO_2_ catalyst was able to convert CH_4_ and CO_2_ into several gaseous products as a function of reaction time (Fig. 7a). Obviously, Au SA/TiO_2_ could produce a significant number of products after 8 hours (around 3800 μmol g^−1^ in total) including H_2_, C_2_H_6_, C_3_H_8_ and CO, with a H_2_ selectivity of about 58%. This performance surpassed that of pure TiO_2_ powder (Fig. S4†) and other reported composites (Table 1). Although the Au SA/TiO_2_ photocatalyst did not exhibit the highest performance in comparison with other composites listed in Table 1, it remains a promising candidate for CH_4_ oxidation reactions.

**Table 1:** Comparison of the methane oxidation performance over the Au SA/TiO_2_ catalyst with other reported composites

Photocatalysts	Amount of catalyst	Reaction conditions	Performance	Ref.
Cu@TiO_2_	10 mg	3 MPa CH_4_	CH_3_OH: 20 000 μmol g^−1^ h^−1^	46
		H_2_O_2_ 1 M 10 mL		
		UV-vis light		
Ru/TiO_2_–H_2_	5.0 mg	8 vol% CO_2_	CO: 708.4 mmol g^−1^ h^−1^, H_2_: 645.5 mmol g^−1^ h^−1^	47
		8 vol% CH_4_		
		84 vol% Ar		
		50 mL min^−1^		
		300 W Xe lamp		
Pt@TiO_2_	2 mg	3 MPa CH_4_	CH_3_OH: 300 μmol g^−1^ h^−1^	48
		300 W Xe lamp		
Rh/TiO_2_	8.6 mg	1% CH_4_	H_2_: 21.5 mmol g^−1^ h^−1^, CO: 21.2 mmol g^−1^ h^−1^	49
		1% CO_2_		
		98% Ar		
		150 W Hg–Xe lamp		
Cr@TiO_2_	10 mg	3 MPa CH_4_	CH_3_OH: 3.45 μmol g^−1^ h^−1^	50
		9.5 mL H_2_O_2_ (30%)		
		Mild conditions		
2% Rh/TiO_2_	20 mg	10% CH_4_	H_2_: 117 μmol g^−1^ min^−1^	51
		3% H_2_O vapor		
		10 mL min^−1^		
		LA-251 Xe lamp, 260 °C		
Au SA/TiO_2_	50 mg	CH_4_ : CO_2_ ∼ 3 : 1	H_2_: 2190 μmol g^−1^	This work
		H_2_O 10 mL		
		300 W Xe lamp		

To determine the role of Au single atoms, the photocatalytic activities of Au SA/TiO_2_ catalysts were compared with those of Au nanoparticle (NP)-based samples. The morphology of typical Au NP-supported TiO_2_ is depicted in Fig. S5.† Under the same reaction conditions, Au SA/TiO_2_ exhibited the highest product yield, outperforming Pt NPs/TiO_2_ and Au NPs/TiO_2_ (Fig. 7b). These results confirm that photocatalytic activities of the single-atom catalyst are more efficient for driving CH_4_/CO_2_ conversion compared to that of nanoparticle-based samples, which could be attributed to its abundant active sites and highly energetic features. Moreover, the effect of Au SA loading on product formation was then investigated. As shown in Fig. 7c, the catalysts with Au SA loading contents in the range of 2–3.5% showed outstanding performances, which were remarkably higher than that of the 0.5% and 5% Au SA loadings. This enhanced performance can be attributed to the improved charge separation rate in the photocatalyst powders.

On top of that, the reactant composition is also a key element and essential to study (Fig. 7d). By varying the pressure ratios of CH_4_ to CO_2_, the photocatalytic yields of the catalyst were changed drastically. Particularly, by feeding CH_4_ and CO_2_ with ratios of 1 : 3 and 1 : 1, the yields of H_2_ and hydrocarbon products were relatively low, while the amount of O-based products increased to around 900 μmol g^−1^ for CO formation, compared to the ratio of 3 : 1, which contains more CH_4_ composition. It is evident that the main products were preferentially generated from CH_4_ rather than CO_2_.

Next, the wavelength-dependent yields were investigated in the visible region and UV region, and under full irradiation for the standard Au SA/TiO_2_ photocatalyst. The catalyst exhibited impressive activities under full illumination and in the UV range, while the visible region was unable to activate CH_4_ and CO_2_ molecules (Fig. 7e). The nonappearance of photocatalytic activities in the visible range could be explained by its deficient energy to excite charge carriers in deep 3d orbitals of the TiO_2_ crystal for CH_4_ activation. As another important factor, the cycling catalytic performance tests were conducted to study the lifetime of the catalyst. As shown in Fig. S6,† there are virtually no changes in the surface morphology of the sample before and after four recycling cycles. The activity of Au SA/TiO_2_ during photocatalytic conversion remained almost stable, with a high yield of 2000 μmol g^−1^ after four cycles (Fig. 7f). A slight decrease in product yield was observed, which was attributed to the reduction of catalyst efficiency during collection and washing processes between cycles.

**Fig. 8:**
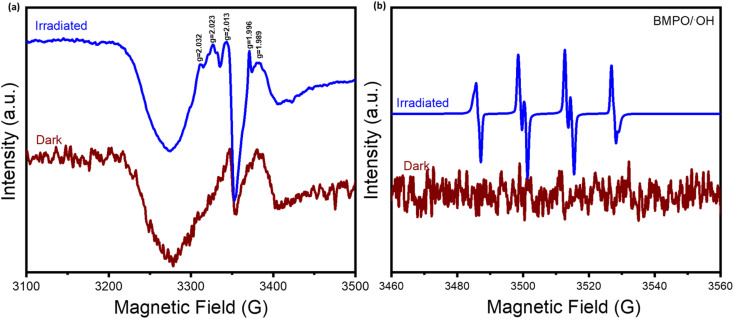
(a) EPR spectra of the Au SA/TiO_2_ sample under subsequential dark and light conditions at 90 K. (b) The spin trapping tests of Au SA/TiO_2_ for detecting hydroxyl (˙OH) radical formation by using 5-*tert*-butoxycarbonyl-5-methyl-1-pyrroline-*N*-oxide (BMPO) as a spin trapping agent.

To elucidate the reaction pathway and mechanism, the electron paramagnetic resonance analysis was carried out. As shown in Fig. 8, unlike under dark conditions, obvious signals of radical oxygen species (ROS) were detected under light irradiation, which are responsible for reaction activation. Importantly, by conducting the spin-trapping test with the BMPO scavenger, the presence of the hydroxyl (˙OH) radical was definitely confirmed, which played an important role in the CH_4_ oxidation process.

Based on the energy band structure and the aforementioned photocatalytic methane oxidation performance, we propose a plausible reaction pathway for CH_4_ on Au SA/TiO_2_. Under dark conditions, CH_4_ adsorbs onto the catalyst surface. Upon illumination, electrons are excited and jump from the valence band to the conduction band, leaving holes behind. As a result, dehydrogenation of CH_4_ occurs on the catalyst surface, forming methyl radicals (˙CH_3_). Furthermore, during the photogenerated charge transfer process, Au plays a crucial role in preventing electron–hole pair recombination, which facilitates the oxidation to OH˙ and H^+^. In subsequent steps, the generated radicals combine with other species to produce H_2_, C_2_H_6_, and C_3_H_8_.^52–54^

## 4. Conclusion

In summary, we have prepared an atomically dispersed Au catalyst supported on TiO_2_ nanostructures with a high loading capacity. Photocatalytic CH_4_ oxidation measurements confirmed that the as-synthesized composite exhibited superior catalytic activity and long-term durability compared to pure TiO_2_ under mild conditions. The main products, including H_2_ and C_2_H_6_, reached a high yield of 2190 μmol g^−1^ with approximately 58% selectivity after 8 hours of operation. We anticipate that this simple approach holds great promise for enhancing single-atom engineering without the formation of nanoclusters.

## Data availability

The data supporting this article have been included as part of the ESI.†

## Conflicts of interest

The authors declare that they have no known competing financial interests or personal relationships that could have appeared to influence the work reported in this paper.

## Supplementary Material

NA-007-D4NA00947A-s001
