# Supplementary material for: Engineering Au single-atom sites embedded in TiO2 nanostructures for boosting photocatalytic methane oxidation
Source: Nanoscale Adv. 2025 Jan 13;7(6):1543–51. doi: 10.1039/d4na00947a (PMC11770591; doi:10.1039/d4na00947a)
Supplement: NA-007-D4NA00947A-s001 [file NA-007-D4NA00947A-s001.pdf]

Supplementary Information (SI) for Nanoscale Advances.
This journal is © The Royal Society of Chemistry 2025

SUPPORTING INFORMATION

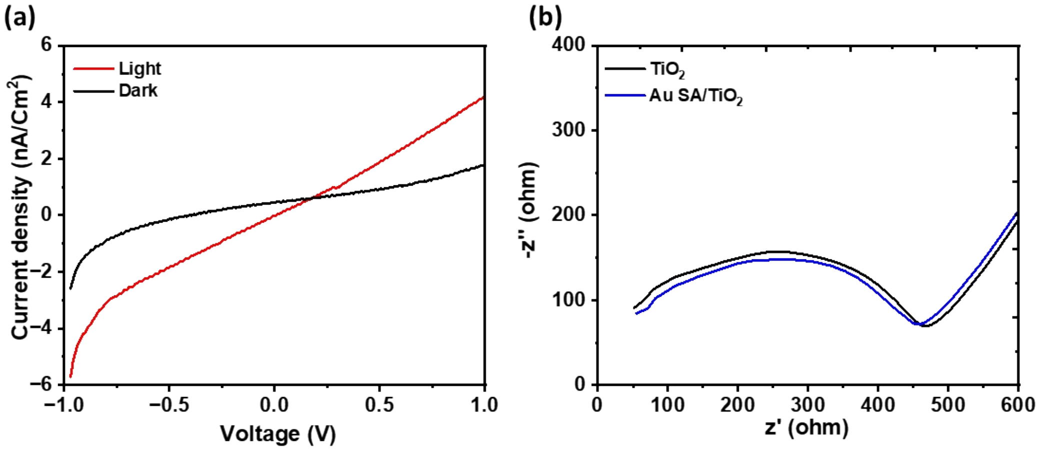

Figure S1. (a) I-V characteristics of Au SA/TiO_2 and (b) EIS Nyquist plots of pure TiO_2 and Au SA/TiO_2 catalysts.

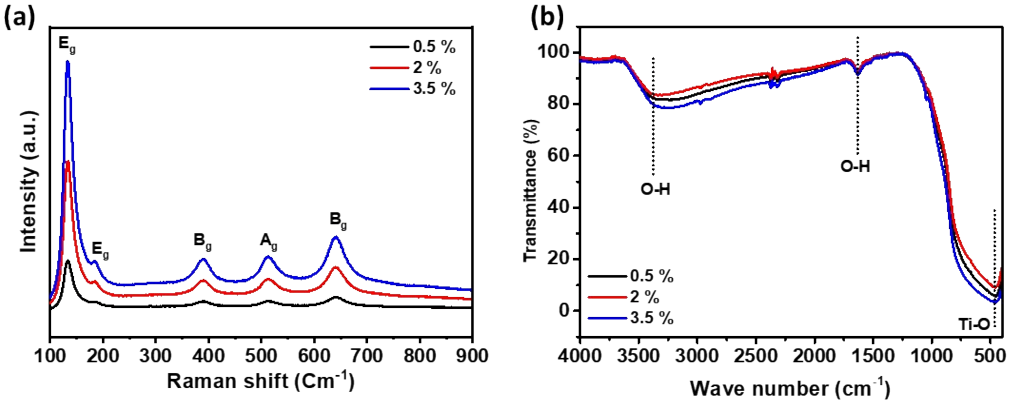

Figure S2. (a) the Raman and (b) FTIR spectrum of Au SA/TiO_2 with different Au loading contents.

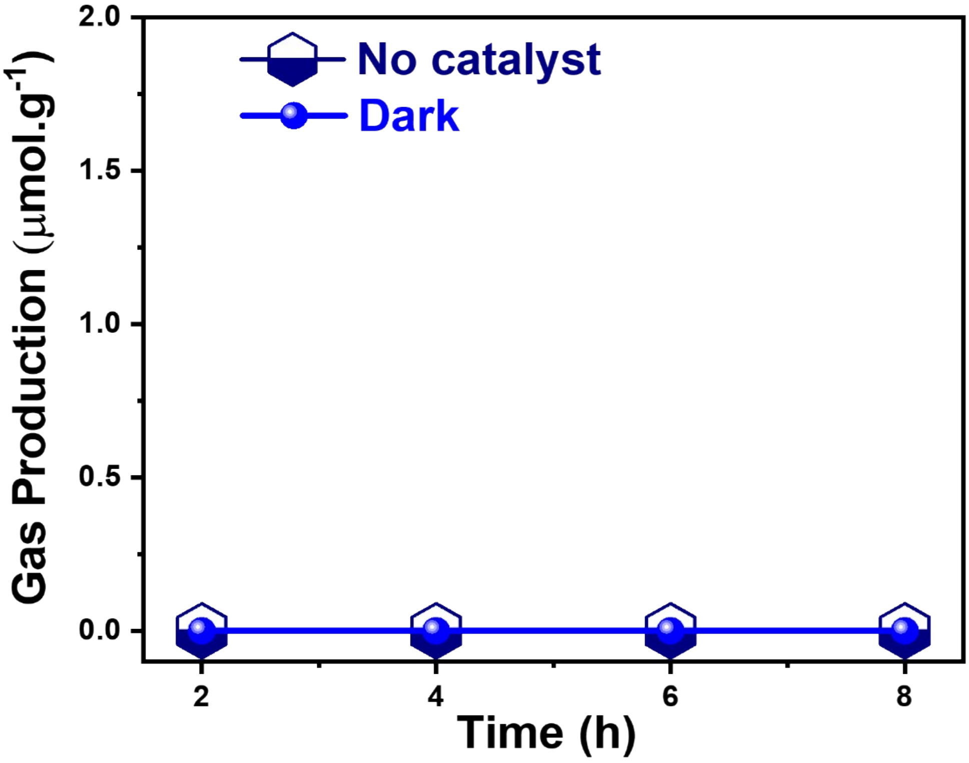

Figure S3. The blank experiments of methane oxidation of Au SA/TiO_2 under different conditions.

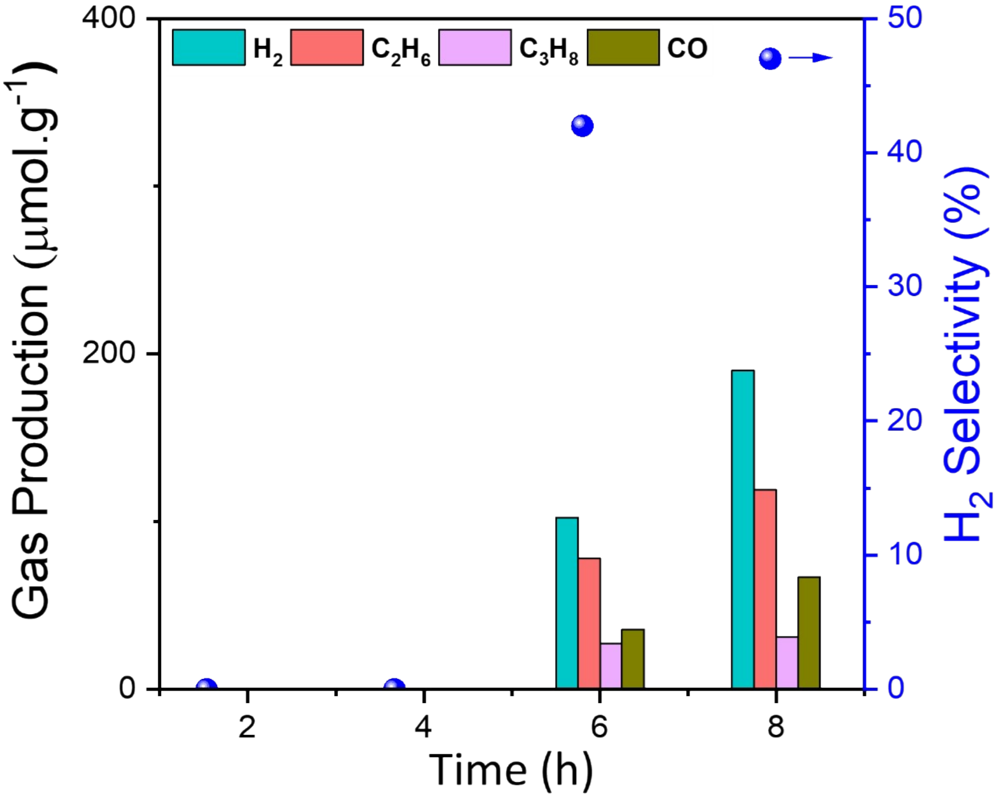

Figure S4. Photocatalytic methane oxidation performances and the H_2 selectivity as a function of reaction time over pure TiO_2. Reaction condition: 50 mg catalyst CH_4: CO_2 = 3 : 1 (stand pressure ratio), Xenon lamp 300W.

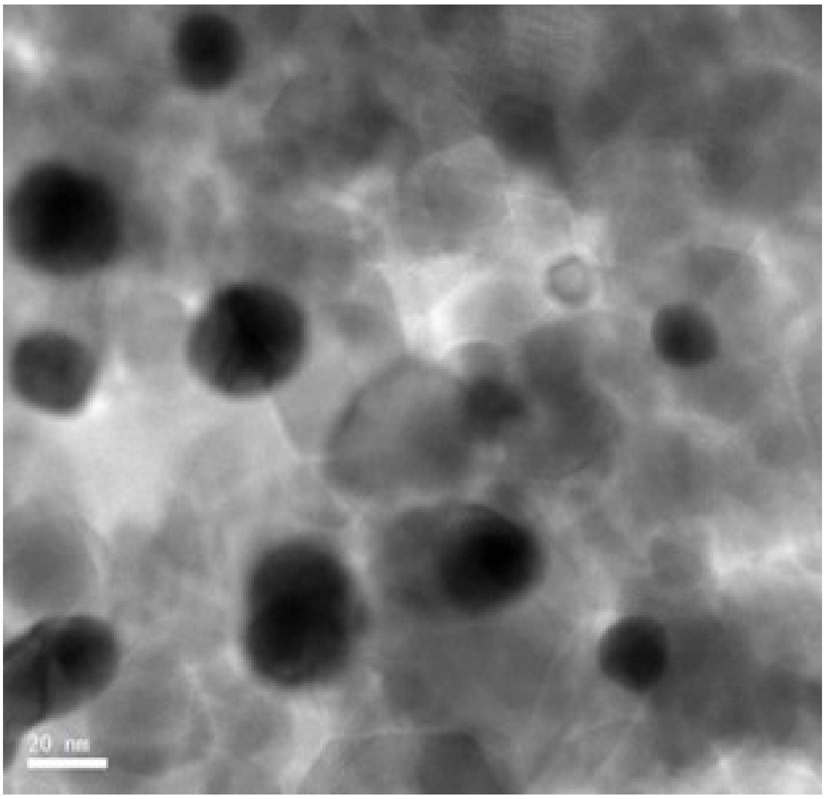

Figure S5. The TEM image of Au NPs/TiO_2.

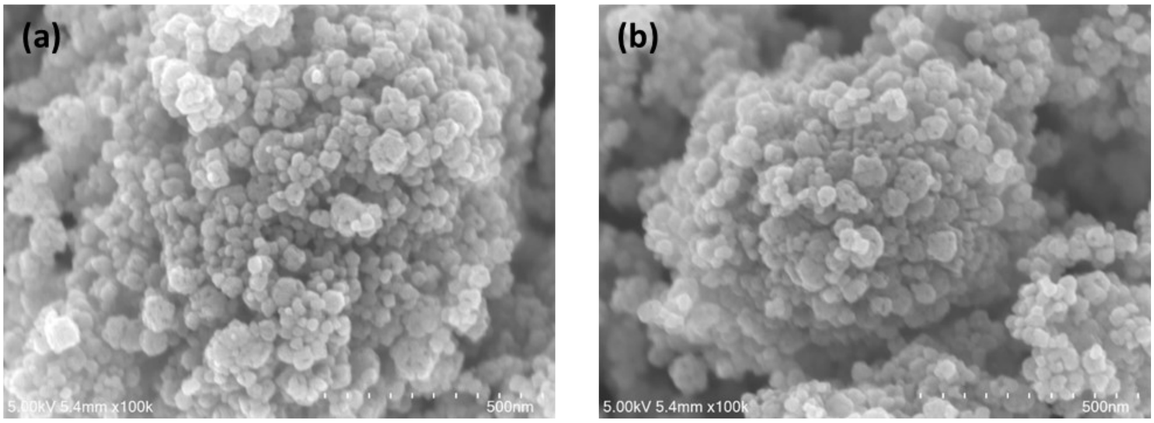

Figure S6. SEM of Au SA/TiO_2 (a) before and (b) after reactions.
